# Transcatheter intervention in a child with scimitar syndrome

**DOI:** 10.5830/CVJA-2016-004

**Published:** 2016

**Authors:** Zhouping Wang, Xiaoyi Cai

**Affiliations:** Department of Cardiology, Guangzhou Women and Children’s Medical Centre, Guangzhou, Guangdong Province, China; Department of Cardiology, Guangzhou Women and Children’s Medical Centre, Guangzhou, Guangdong Province, China

**Keywords:** scimitar syndrome, congenital heart disease, transcatheter intervention

## Abstract

Scimitar syndrome is a rare congenital heart disease characterised by anomalous pulmonary venous drainage to the inferior vena cava, aortopulmonary collaterals, hypoplasia of the right lung and intracardiac defects. Surgical correction remains the gold-standard therapy. However, non-surgical intervention has been reported effective in selected cases with scimitar syndrome. We report on a one-year-old boy with scimitar syndrome who underwent stepwise transcatheter intervention as an alternative treatment. Embolisation of the aortopulmonary collaterals and occlusion of the atrial septal defect were performed using detachable coils and an Amplatzer septal occluder, respectively. The patient’s postcathetherisation course was uneventful. The right cardiac chamber and pulmonary arterial pressure returned to normal during follow up.

## Abstract

Scimitar syndrome is a rare congenital anomaly consisting of anomalous pulmonary venous drainage from the right lung to the inferior vena cava (IVC), hypoplasia of the right lung, cardiac dextroposition, and malformation of the right pulmonary artery and aortopulmonary collaterals (APC) supplying the lower lobe of the right lung.^1^,^2^,^3^ Its prevalence is low and is estimated at two out of every 100 000 live births.^4^

The clinical presentations are quite diverse, ranging from severe congestive heart failure in infancy to mild symptoms in childhood, and depend greatly on the extent of left-to-right shunting from partially anomalous pulmonary venous drainage and aortopulmonary collateral flow.^5^,^6^ Scimitar syndrome has two types. One is the adult form, which is usually asymptomatic and not associated with other cardiac disorders. The other type is the infantile form, presenting with signs and symptoms of severe pulmonary hypertension, associated cardiac malformations, and large systemic collateral arteries feeding the right lung in the first weeks of life. Transcatheter embolisation of the APC and repair of co-existing cardiac defects may improve symptoms and decrease pulmonary arterial pressure in symptomatic patients.^7^,^8^

Herein we describe a boy with scimitar syndrome who had obvious clinical improvement and pulmonary arterial pressure drop after transcatheter embolisation of the APC and atrial septal defect closure.

## Case report

A one-year-old, 9.5-kg male was admitted to our hospital (Guangzhou Women and Children Medical Centre, China) with tachypnoea. He had a history of recurrent pneumonia, sweating and growth retardation.

**Fig. 1:**
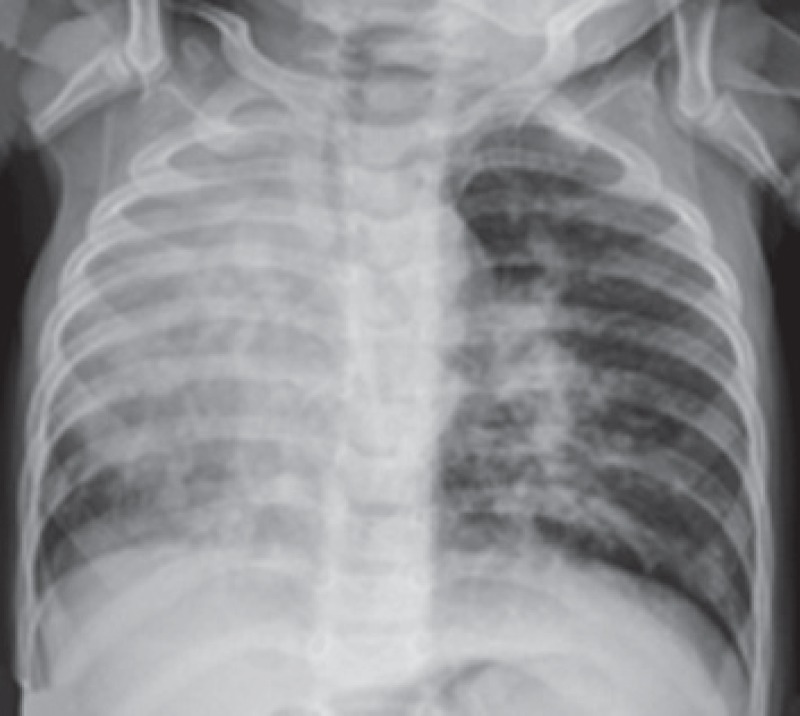
Chest X-ray showing cardiac dextroposition and radiopaque right hemithorax.

On admission, a physical examination revealed grade II/VI systolic murmur at the left sternal border. Moreover, cardiac dextroposition and a radiopaque right hemithorax were confirmed on a chest X-ray examination (Fig. 1). Echocardiography showed an enlarged right cardiac chamber, partially anomalous pulmonary venous drainage, an 8-mm secundum atrial septal defect (ASD). The systolic pulmonary arterial pressure was 63 mmHg, calculated from tricuspid regurgitation velocity.

**Fig. 2:**
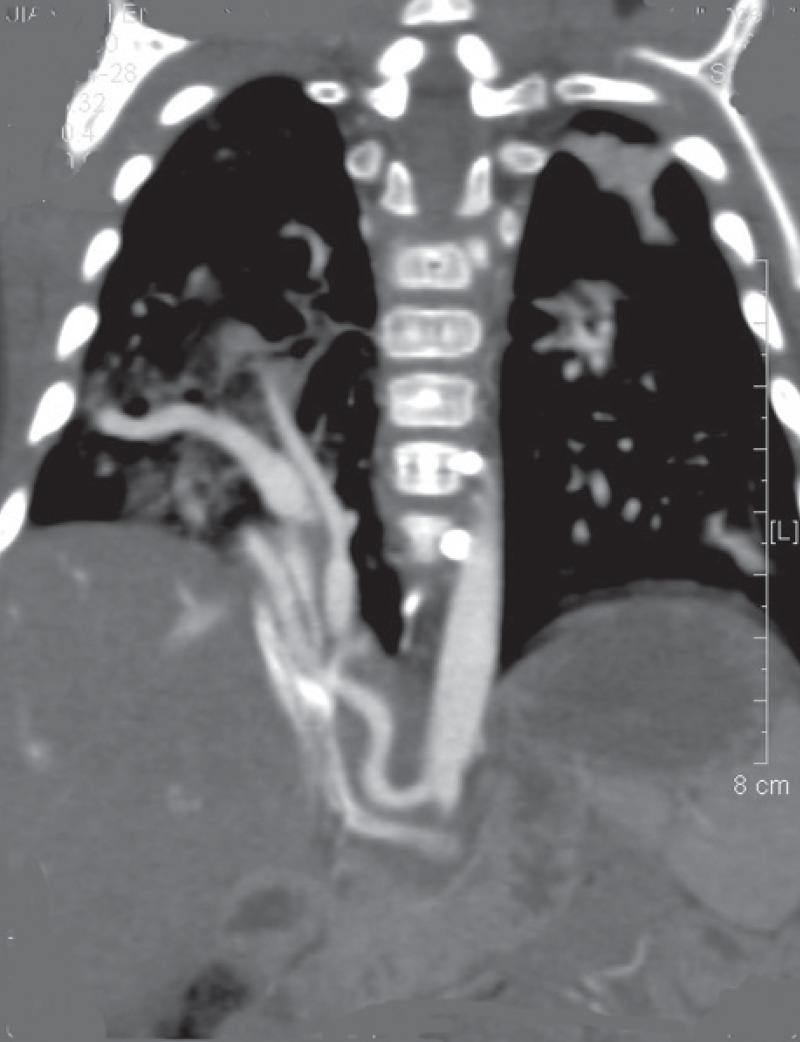
CTA showing right pulmonary vein drainage to the inferior vena cava.

The computerised tomography angiography (CTA) revealed partially anomalous pulmonary venous drainage to the IVC, APC originating from the abdominal aorta, and hypoplasia of the right lung (Fig. 2), which confirmed the diagnosis of scimitar syndrome. The patient’s parents refused conventional surgical intervention, considering the high risk of postoperative events.

**Fig. 3:**
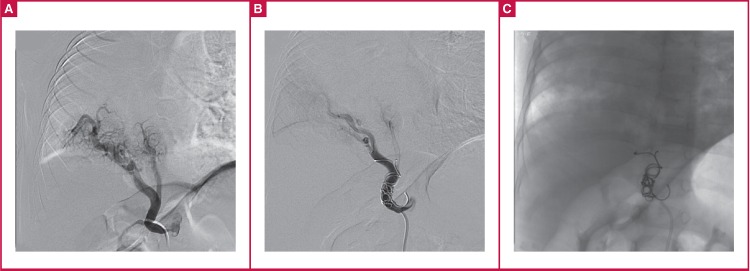
(A) Catheterisation showing systemic arterial collaterals arising from the abdominal aorta and supplying the right lung. (B) and (C) Catheterisation showing coil embolisation of the systemic arterial collaterals supplying the right lung.

As an alternative, stepwise transcatheter intervention and coil embolisation of the APC and closure of the ASD were selected to decrease the left-to-right shunt and reduce pulmonary arterial hypertension. The patient proceeded to have cardiac catheterisation under general anaesthesia. A 4-F Cobra cathether (Cook, Bloomington, IN) was delivered to the APC via the femoral artery and then two 8 × 50-mm MReye embolisation coils (Cook, Bloomington, IN), five 5 × 30-mm MReye embolisation coils and two 5 × 30-mm Cook coils (Cook, Bloomington, IN) were deployed for occlusion (Fig. 3A,B).

**Fig. 4:**
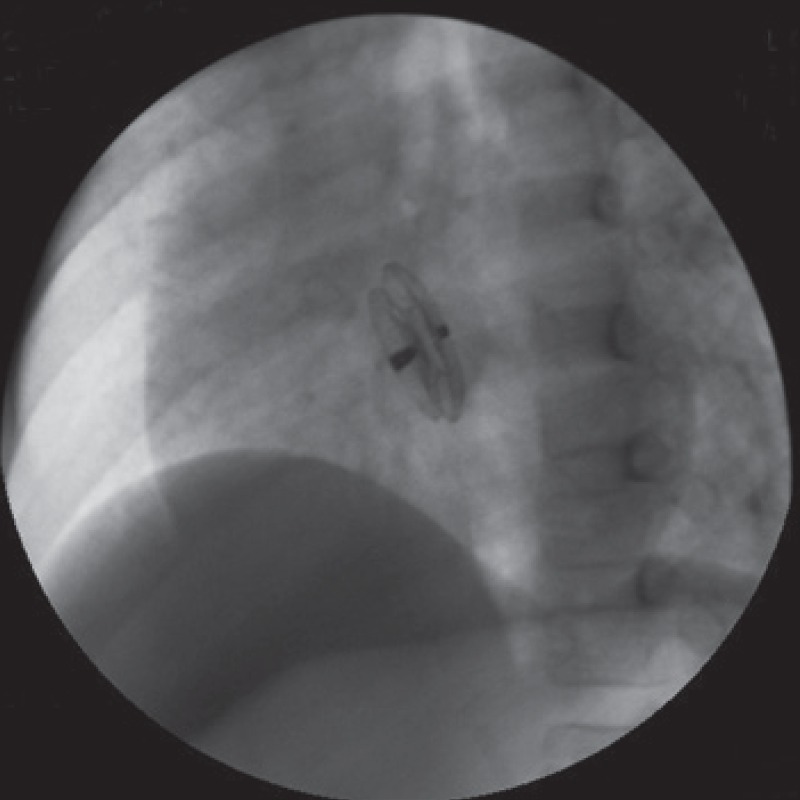
Catheterisation showing complete occlusion of the atrial septal defect by the Amplatzer septal occluder.

After percutaneous entry of the femoral vein, a sizing balloon 8-F MPA2 catheter was introduced over an extra-stiff guide wire positioned in the left upper pulmonary artery to measure the pulmonary arterial pressure (pathway: IVC->RA: right atrium->RV: right ventricule->MPA: main pulmonary artery). Using an exchange 260-cm Cordis guidewire, an occlusion balloon catheter was introduced into the left atrium to determine the stretched diameter of the ASD (pathway: MPA->RV->RA->ASD->LA). A 10-mm Amplatzer septal occluder (AGA Medical, MN) was implanted after balloon sizing the defect (diameter: 8 mm) (Fig. 4). The procedure was successful.

The total surgical time was 150 min; fluoroscopy time was 45 min. Pulmonary-to-systemic flow ratio (Qp/Qs) dropped from 2 to 1.2 and pulmonary arterial pressure decreased from 70/41 (50) mmHg to 45/14 (24) mmHg.

After intervention, the patient improved clinically and was treated with asprin and frusemide. Three months later he was asymptomatic, and he was clinically well at the 12-month follow-up assessment. The indicators of right cardiac chamber and pulmonary arterial pressure estimated by echocardiography were normal.

## Discussion

Scimitar syndrome has a wide complex of anomalies, including drainage of all or part of the right lung to the inferior caval vein, hypoplasia of the right lung, pulmonary hypertension, dextroposition of the heart and anomalous systemic arterial supply.^9^ The anomaly was first described by Cooper in London in 1836 during autopsy of an infant.^4^

The aetiology of scimitar syndrome is not clear. Clinical presentation of patients with scimitar syndrome depend on the degree of pulmonary arterial hypertension, which is often secondary to left-to-right shunt from the pulmonary vein, co-existing intracardiac shunt, pulmonary hypoplasia, resulting in reduction of the vascular bed, and systemic arterial supply to the right lung.

A surgical approach has been recognised as the goldstandard therapy for scimitar syndrome, including rerouting of the aberrant pulmonary vein and repair of the other cardiac defects.^2^,^10^ However, a two-staged strategy with catheter-based embolisation of the APC, followed later by correction of the anomalous veins, has also been recommended in some cases. There is no consensus on which is the best option.

Recently, multiple reports have shown transcatheter intervention, including embolisation of the APC and closure of the cardiac defects, may improve symptoms, decrease pulmonary arterial pressure,^11^,^12^ and postpone or even eliminate the need for surgical correction.^7^,^8^,^13^ Instead of a surgical approach, patients with scimitar syndrome who have significant left-to-right shunt due to APC and other cardiac defects are largely suitable for transcatheter intervention.

In our case, the patient’s pulmonary arterial hypertension was attributed to the ASD and APC, which could be improved by decreased pulmonary arterial pressure after transcatheter intervention. We opted for embolisation of the APC and closure of the ASD. The patient had satisfactory clinical improvement and no surgery was performed during follow up. No pulmonary infarction was observed during the follow-up period.

Many kinds of devices, such as detachable coils, AVPII and AVPIV have been reported to be appropriate for embolisation of the APC. In this case, we used detachable coils due to their ease of deployment, extractability, small size of catheter required, low metal content and affordability

Besides the therapies mentioned above, others have also been used in scimitar syndrome. Pneuomonectomy or lobectomy is performed cautiously in patients with persistent haemoptysis, thrombosed intra-atrial baffle, obvious hypoplasia of the right lung,^14^ or right pulmonary vein obstruction at risk of scoliosis post operation^2^, and respiratory insufficiency post pneuomonectomy.^10^,^12^,^15^

## Conclusion

Although the traditional surgical approach is still the gold standard in symptomatic patients, transcatheter intervention of embolisation of the aortopulmonary collaterals and repair of co-existing cardiac defects may be an option for selected patients with scimitar syndrome.
